# Multi-dimensional impacts of Coronavirus disease 2019 pandemic on Sustainable Development Goal achievement

**DOI:** 10.1186/s12992-022-00861-1

**Published:** 2022-06-27

**Authors:** Angkana Lekagul, Anamika Chattong, Putthipanya Rueangsom, Orratai Waleewong, Viroj Tangcharoensathien

**Affiliations:** International Health Policy Program, Ministry of Public Health, Nonthaburi, 11000 Thailand

**Keywords:** COVID-19, Sustainable Development Goals, SDGs, Causal loop diagram, Impact pathway

## Abstract

**Background:**

Health, social and economic crises triggered by the Coronavirus disease pandemic (COVID-19) can derail progress and achievement of the Sustainable Development Goals. This commentary analyses the complex nexus of multi-dimensional impacts of the pandemic on people, prosperity, planet, partnership and peace. From our analysis, we generate a causal loop diagram explaining these complex pathways and proposed policy recommendations.

**Main text:**

Health systems, health and wellbeing of people are directly affected by the pandemic, while impacts on prosperity, education, food security and environment are indirect consequences from pandemic containment, notably social measures, business and school closures and international travel restrictions. The magnitude of impacts is determined by the level of prior vulnerability and inequity in the society, and the effectiveness and timeliness of comprehensive pandemic responses.

**Conclusions:**

To exit the acute phase of the pandemic, equitable access to COVID-19 vaccines by all countries and continued high coverage of face masks and hand hygiene are critical entry points. During recovery, governments should strengthen preparedness based on the One Health approach, rebuild resilient health systems and an equitable society, ensure universal health coverage and social protection mechanisms for all. Governments should review progress and challenges from the pandemic and sustain a commitment to implementing the Sustainable Development Goals.

## Background

In September 2015, the United Nations (UN) member states adopted the 2030 Agenda for Sustainable Development (SDGs) [1]. Based on three interlinked strategic pillars of development, social, economic, and environmental, the SDGs are a comprehensive development blueprint geared towards peace, prosperity, people, and the planet through collaborative partnership. Unlike the Millennium Development Goals, the SDGs are for all countries at all levels of development (low-, middle- and high-income countries) and all stakeholders. UN member states advocated “leave no one behind” as the central promise to eradicate poverty and exclusion and reduce inequalities and vulnerabilities.

Excerpts from the 2019 UN SDG report, Table 1, show uneven progress and remaining challenges across geographical regions in achieving the 17 goals prior to COVID-19 pandemic. Countries in sub-Saharan Africa and Southern Asia have made the least progress across many targets. The slow implementation of SDGs brought together international partners to reaffirm their commitments, through pledging of the “SDG Decade of Action” in 2020.

**Table 1:** SDG achievement in 2019 prior to Pandemic

SDG1: The world is on track to end poverty by 2030; however, 55% of world’s population have no access to social protection; 736 million people lived in extreme poverty in 2015 in which more than a half living in sub-Saharan Africa. SDG2: Two thirds of extremely poor employed workers worldwide are agricultural workers; 821 million were undernourished in 2017 up from 784 million in 2015; Two thirds of undernourished people worldwide live in two regions: sub-Saharan Africa and South Asia. SDG3: Under five mortality drops from 9.8 million in 2000 to 5.4 million in 2017; tuberculosis incidence rate declined by 21% between 2000 and 2017; vaccinations resulted in an 80% drop in measles deaths between 2000 and 2017 SDG4: 617 million children and adolescents lack minimum proficiency in reading and mathematics while 750 million adults still remain illiterate; two thirds of them are women. SDG5: 18% of ever-partnered women and girls aged 15 to 49 years have experienced physical and/or sexual partner violence in the previous 12 months though In Southern Asia, a girl’s risk of marrying in childhood has decreased by 40% since 2000. SDG6: In 2017, 785 million people remain without even basic drinking water service; and 2 out of 5 people worldwide do not have a basic handwashing facility with soap and water at home (2017). SDG7: 9 out of 10 people worldwide have access to electricity for which 87% of 840 million people without electricity live in rural areas. SDG8: Real Gross domestic product (GDP) grew by 4.8% annually in Least Developed Countries (LDC) (2010–2017), less than the 7% SDG target; median hourly pay of men is 12% higher than that of women. SDG9: Industrialization in LDCs is too slow to meet the 2030 Agenda target; Manufacturing value added per capita in LDC (US$ 114) is far lower than in Europe and northern America (US$ 4,938) SDG10: In many countries, an increasing share of income goes to the top 1% while the bottom 40% receive less than 25% of overall income SDG11: 1 out of 4 urban residents live in slum-like conditions; 2 billion people do not have access to waste collection services; SDG12: The global material footprint is rapidly growing, outpacing population and economic growth; material footprint per capita in high-income countries is 60% higher than in upper-middle income countries and more than 13 times the level in low- income countries. SDG13: The global mean temperature in 2018 is approximately 1°C above the pre-industrial baseline. Atmosphere CO_2_ concentration is 146% of pre-industrial levels. SDG14: Ocean acidity has increased by 26% since pre-industrial times. The proportion of fish stocks within biologically sustainable levels declined from 90% (1974) to 67% (2015). SDG15: The risk of species extinction has worsened by almost10% over the last 25 years. SDG16: Men make up around 80% of homicide victims overall but women constitute 64% of homicide victims of intimate partner/ family-related homicide SDG17: Net Official Development Assistance totalled $149 billion in 2018, down by 2.7% from 2017; Over 80% of people in developed countries are online compared to 45% in developing countries and only 20% in least developed countries.	

Source: Sustainable Development Goals Report 2019 [2]

Coronavirus disease 2019 (COVID-19), caused by SARS-CoV-2 emerged in late 2019 and has continued into 2022 with devastating impacts. By 31 January 2022, there were 385 million infections and 5.7 million deaths worldwide [3]. The pandemic was further complicated by the emergence of variants of concerns such as the Delta variant in the middle of 2021 and Omicron variant at the end of 2021. These variants can reduce the effectiveness of vaccines, especially the Delta variant [4].

The transmission dynamic in Brazil, one of the fast-growing epidemics in the world, suggests non-pharmaceutical interventions are effective during the initial phase in reducing the reproduction number of the virus, but lack of international travel restriction resulted in more than 75% of viral strains in Brazil being introduced from Europe that finally propagated through local transmission across states and major urban centres [5].

The pandemic lasted for two full years in 2020 and 2021 with no sign of recovery in 2022 due to the Omicron variant. Low- and middle-income countries rely on a vaccine-sharing arrangement called COVAX, which aimed to deliver two billion doses by the end of 2021. However, COVAX faced interruptions from production bottlenecks, export bans and wealthy nations securing more vaccine than they needed through advanced purchase agreements. While only 10% of populations in low-income countries have received at least one dose, 93.5% of the United Arab Emirates population are fully vaccinated [6]. Low vaccine coverage allows continued circulation of the coronavirus, and the emerging variants can be spread worldwide through international travel.

The pandemic came right in the middle of SDGs implementation. With its extraordinary consequences, the pandemic can reverse past gains and hamper SDGs achievement by 2030. This commentary examines the extent of pandemic impacts in a comprehensive manner by covering all three pillars of sustainable development: economic, social, and environment, and proposes policy recommendations on resilient pandemic recovery and maintenance of sustainable development momentum towards 2030.

**Fig. 1:**
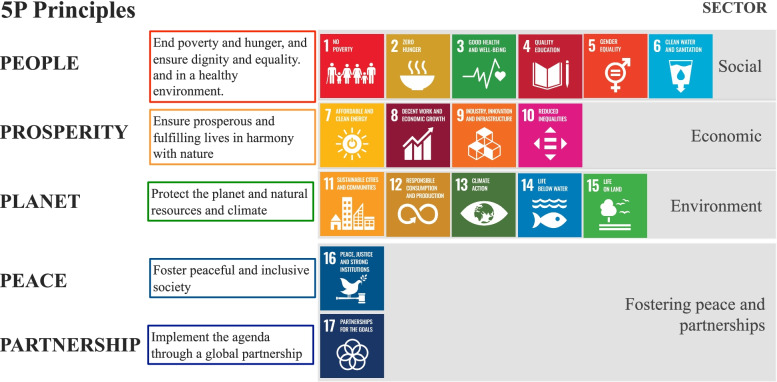
Interlinks between 17 SDGs and the 5P principles: people, prosperity, plant, peace and partnership. Source: Modified from SDG Services [7]

At the time of preparing this manuscript (middle of 2021); a systematic review is not feasible as there was no full assessment of pandemic impacts on SDGs. Few literatures reported actual impact, while several reported plausible impacts or demonstrate pathways of possible impacts such as food security, delayed in learning due to school closures. Hence, we conducted exploratory review of published literatures which reported the impacts of COVID-19 pandemic on SDGs at regional and global levels. We used evidence from these papers as inputs for analysis of pandemic as a cause which resulted in either positive or negative impacts on various SDGs. Impacts presented in these literatures were either plausible or actual impacts which were supported by quantitative evidence. The authors then generated the cause and outcome relationship and its cascades of cause and outcomes using Vensim®. As the 17 SDGs are interlinked, indivisible and focus efforts in five “P” areas, namely people, planet, prosperity, peace and partnership [1]; impacts demonstrated in the article were synthesised by the five “P” areas. Figure 1 depicts interlinks between 17 SDGs and the five Ps.

### Pathways of impacts on SDGs

**Fig. 2:**
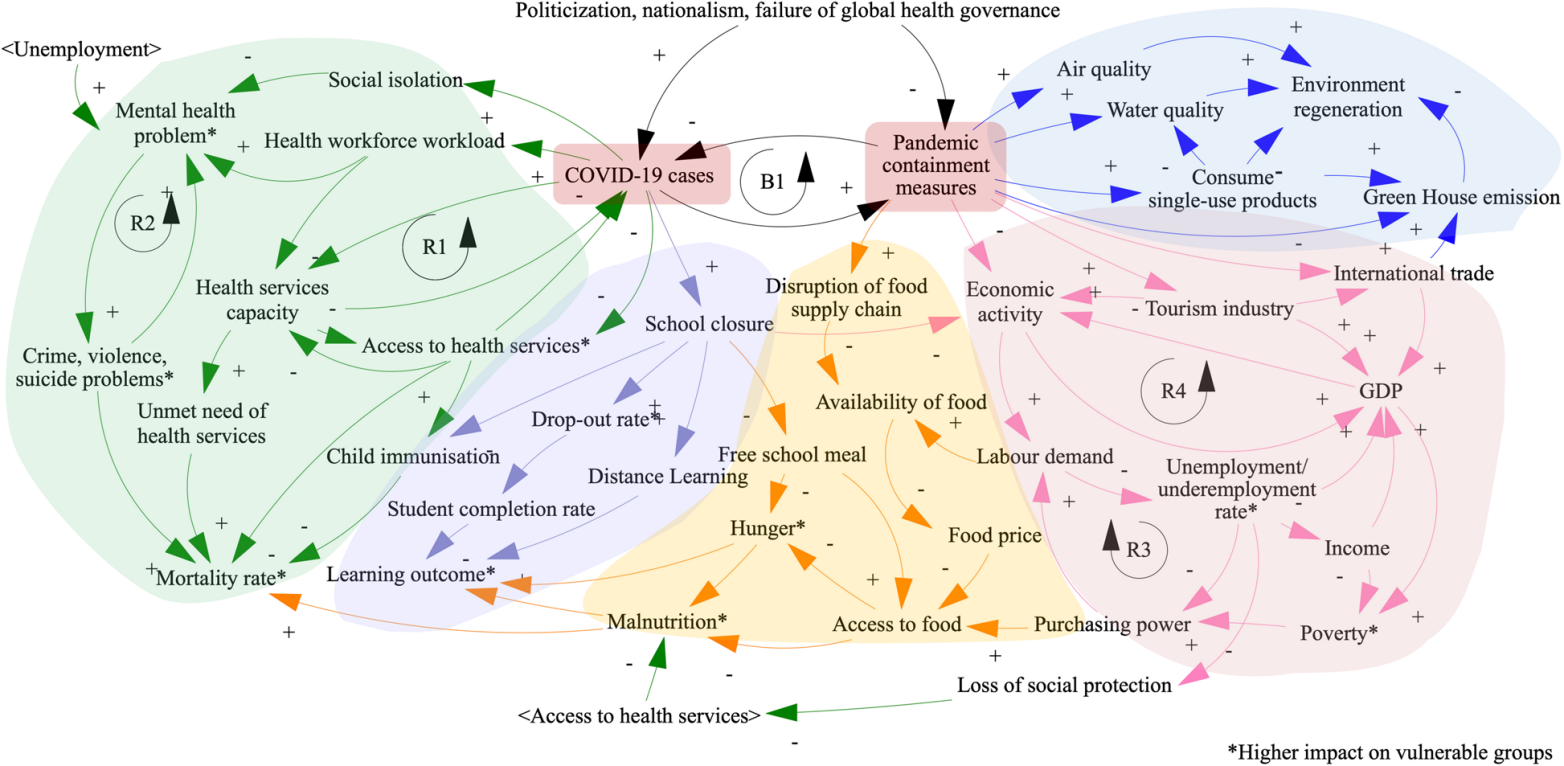
A causal loop diagram demonstrating the COVID-19 impact on different aspects of SDGs: health, education, food systems, economy, and the environment. Note: Coloured background reflects five areas of pandemic impacts: health (green), education (navy blue), economics and employment (pink), environment (royal blue) and food and nutrition (orange)

One cause can have various effects, and in turn, these effects become the causes of the next generation of effects. To understand the complex nature of multiplications and cascades of impacts, we constructed a causal loop diagram to demonstrate the interrelationships of causes and effects of the COVID-19 pandemic [8] by using the Vensim® software programme. In the causal loop diagram as shown in Fig. 2, a positive or negative sign is labelled at the tip of arrow to demonstrate the nature of impacts from the pandemic. The five colours denote pandemic impact on five groups of SDGs, namely a) health (green), education (navy blue), economics and employment (pink), environment (royal blue) and food and nutrition (orange).

In Fig. 2, there are two major origins of determinants. First, the pandemic itself has substantive negative impacts on the health of populations and on education due to school closures. Second, national and international travel restrictions were implemented as major social measures to contain the pandemic; such restrictions have significant negative impacts on the economy and employment. Movement restrictions have both positive and negative impacts on environmental regeneration. Both origins (the pandemic itself and movement restrictions) and their cascade consequences have synergistic negative impacts on access to food and nutrition. An asterisk (*) denotes higher adverse impact on the vulnerable population.

### Impact of the pandemic on people

Infections and deaths are concentrated among the vulnerable populations, ethnic minorities and the poor. A meta-analysis showed that ethnic minorities had greater risk of COVID-19 infection and mortality [9]. Though age, poverty, obesity, diabetes, hypertension and cardiovascular diseases are key risk factors among United States ethnic minorities, when these multicollinear risks are removed, poverty stands out as key risk factor for mortality [10]. The long-standing societal inequality in the United States and the living and working conditions among minority communities result in increased risk of COVID-19 mortality [11]. In the African American community, coronavirus infection and mortality are driven by interactions of environmental, social and biological factors [12]. Socio-economic factors are also key drivers of variations in COVID-19 morbidity and mortality in the UK [13, 14].For example, the Pakistani descendants in UK are three times more likely than White British to live in the most deprived neighbourhoods [15]. A systematic review suggested Black Asian and Minority Ethnic individuals had increased risk of and worse clinical outcomes from COVID-19 when compared to White individuals [16].

Approximately one million excess deaths occurred in 2020 among all 29 high-income countries except for in New Zealand, Norway, and Denmark. In almost all countries, age-standardised excess death rates were higher in men than women, which widens the gender inequality of mortality [17].

New Zealand’s comprehensive and timely response, an evidence-based and science-led approach prioritised on protecting lives, effective communication, and leadership style are major contributing factors of effective responses [18]. A study across 175 countries shows that cancelling public events, imposing restrictions on private gatherings and closing schools and workplaces had significant effects on reducing COVID-19 infection [19].

The provision of essential health services was interrupted as health workers were mobilised to support pandemic containment. Hospitals have postponed services to minimise risk of infection while travel restrictions prevent patients from seeking the needed care. We identified two reinforcing feedback loops (Fig. 2). R1 shows that surge of COVID-19 cases diminished health service capacity, preventing delivery of other essential health services, and increasing COVID-19 mortalities due to depletion of essential resources such as ICUs and ventilators. Reduced health service capacity resulted in high unmet healthcare need and caused high mortality from non-COVID-19 causes. The disruption of essential health services included primary health care services [20], gynaecology services [21], immunisation [20, 21], treatment of non-communicable diseases [21–23], cancer screening [20], sexual and reproductive health services [20, 22, 24, 25] and mental health services [23, 25, 26]. Childhood vaccine coverage declined due to global supply-chain disruptions [21]. In addition, the pandemic increased unmet need for family planning by 40% and increased unintended and teenage pregnancies significantly [20, 21, 25]. The World Health Organisation, in its second pulse survey in 2021, reported that on average 38% of overall 63 tracer services have been disrupted by the pandemics [20].

The pandemic also resulted in deterioration of health service quality [24] and deployment of health workers for pandemic responses. Maternal and child outcomes have worsened with an increase in maternal and child deaths [21, 22, 25–27]. Estimates showed an additional 8.3 to 38.6% increase in maternal mortality ratio in LMICs [22]. Studies have also reported negative mental health impact, increased alcohol and substance use, and increased psychological stress [23, 24]. Moreover, health workforce stress from heavy workload related to pandemic responses have led to mental health problems and other psychological consequences [28].

In addition, COVID-19 disrupted education systems due to resulting school closures, causing poorer households to shoulder higher burdens due to lower capacity for online learning [24, 26]. Dropout rates increased and school completion rates decreased, leading to low levels of learning outcomes. Provision of essential services such as school meals and vaccination for children were interrupted [22, 23, 26, 27]. It is estimated that 379 million children were affected from lack of school meal provision globally [22]. UN Economic and Social Commission for Asia and the Pacific reported that 850 million students in Asia Pacific lost almost half their academic year by 2020 and 6.7 million students dropped out of school [25]. Even though schools provided online learning, vulnerable students in particular have difficulties in accessing remote learning due to lack of computers and internet facilities [21, 23]. For instance, 20% of the students in East Asia and the Pacific Region and 38% of the students in South and West Asia do not have access to remote learning [25]. Decreased government expenditure on education further complicates the situation [21, 23, 26].

The World Food Programme estimates that in 2021, 296 million people in 35 countries received insufficient food, an increase of 111 million more people compared to 2020 [29]. Figure 2 shows how low food availability due to distribution restrictions leads to higher food prices, and when combined with job losses increases household risk of food insecurity [23, 27, 29]. Food prices have reportedly increased by 10–20% particularly in import-dependent countries [27]. UN reported that about 265 million people faced hunger and food insecurity and an additional 10 million children were malnourished in 2020 [21]. Children under 5 years of age were severely affected by acute undernutrition (wasting) and chronic undernutrition (stunting) [22]. In 2022, this was estimated to be 6.7% wasting (45.4 million children) and 22% stunting (149.2 million children) [29]. In the United Kingdom, food poverty significantly increased due to lack of access to free school meals during school closure [26]. Income reduction from underemployment or unemployment further reduce the purchasing power for food, leading to undernutrition particularly in poor and vulnerable populations [24]. Moreover, people have redirected their purchasing behaviours towards longer shelf life and poorer nutrition foods which exacerbates undernutrition [27].

### Impact of the pandemic on prosperity

In 2021, although the world economy witnessed an exceptionally strong recovery globally, with about 6% GDP increase, the 2022 recovery is predicted to be very uneven, underpinned by steady but highly unequal vaccine access across countries. Growth is concentrated in a few major economies while most of emerging markets and developing economies lag behind. For example, 90% of advanced economies will regain their pre-pandemic per capita income by 2022, but only about one-third of the emerging markets and developing economies will be able to do so. The worst economic prospects are among low-income countries, with a foreseen reverse gain in poverty reduction. This is compounded by food insecurity and other long-standing challenges [30].

In Fig. 2, the feedback loop R4 demonstrates that low economic activity reduces labour demand, leading to increased unemployment and underemployment rates and decreased household incomes which subsequently contribute to low or negative GDP growth. Economic activity includes both exportation and importation, and both demand and supply chains such as those of food processing industries and retailers. The protracted recession could progress towards depression [31].

Most countries reported negative GDP growth and increased debt-to-GDP ratio as governments take out loans for pandemic responses and vaccine procurement [22, 27, 29, 32, 33]. Travel restrictions have major negative impacts in countries with a high share of GDP coming from tourism and service industries. World Economic Situation and Prospects reported that the tourism industry was hit with the grounding of 90% of fleets [32]. The pandemic also caused a significant drop in international trade including reductions in foreign direct investment and global merchandise trade [22].

R3 highlights the reinforcing loop between reduced demand for labour and increased unemployment and its cascade impacts. Due to the travel restrictions and a fall in economic activity, labour market shock resulted in significant reductions in working hours and increased underemployment, wage cuts, and unemployment. The ILO estimated labour income loss at about US$ 860 and US$ 3,440 billion due to COVID-19 [32]. World Bank estimated an increase in extreme poverty, as a result of job and income loss exacerbated by lack of effective government social protection mechanisms [33].

Similarly, some reports showed an increase in unemployment rates (more than 11% in United Kingdom, and 3.1% in Thailand) [24, 26]; in particular, higher unemployment among women and young workers [21, 25]. In Thailand, undocumented migrant workers and some informal sector workers do not have social security coverage to healthcare and do not get paid sick leave [24]. The global poverty headcount is estimated to increase by as much as 100 million people [21]. At the micro-level, effects on household are clear—reduced income and increased debt. Several vulnerable populations risk homelessness from inability to pay rent for a room. The debt burden is even more severe among low-income households, agricultural families, and older persons with inadequate social protection [24].

### Impact of the pandemic on planet

On planetary health, the environment is the cause of and has braced both negative and positive effects from COVID-19. COVID-19 is a result of the growing threat from zoonosis and emerging infectious diseases, which continue to rise with the invasion to wildlife habitats by human activity and subsequent increased human exposure to new pathogens [34].

A study in Italy reported the correlation between air pollution and the number of COVID-19 infections. More than 75% of infected people and about 81% of mortality from COVID-19 were in industrialized areas with high levels of air pollution (> 100 days per year exceeding the limits set for PM10 [35, 36]. Other studies demonstrated that warmer temperature and moderate outdoor ultraviolet exposure resulted in a slight reduction in the transmission of SARS-CoV-2 [37]. The wind speed of two metres per second had a significant positive correlation with COVID-19 cases [38]. High levels of PM2.5, NO2 and precipitation were positively associated with COVID-19 mortality in Spain [39]. Average temperature, minimum temperature, and air quality were significantly associated with the COVID-19 pandemic in New York [40].

Due to travel restrictions, global daily CO_2_ emissions dropped by 17% in April 2020 when compared to April 2019 [25]. A significant reduction in air pollution as measured by PM2.5 (fine particulate matter of 2.5 µm or less in diameter, should not exceed 5 µg/m^3^, while 24-h average exposures should not exceed 15 µg/m^3^ more than 3—4 days per year [35]) concentrations was observed in India (50%), China (34%), and some cities in South-East Asia such as Bangkok, Kuala Lumpur, Manila and Singapore [25]. The water quality also improved in many countries [21]. Lockdown measures allow free movement of wildlife and contribute to environmental regeneration. Plants and animals reclaimed space, including endangered species that gradually recovered in protected areas in both forests and sea [21, 25].

However, the pandemic increased significant demand for and disposal of single-use plastic bags, mask packaging and used face masks amongst people, and personal protective equipment for health personnel. It resulted in gigantic volumes of plastic and medical waste. Wuhan, China reported a massive increase of daily medical waste from about 40–50 tons in 2019 (before the outbreak) to approximately 247 tons in 2020 [25]. Cities such as Bangkok, Hanoi, Kuala Lumpur and Manila reported similar rises, generating 154–280 tons more medical waste per day than before the pandemic [25].

### Impact of the pandemic on peace and partnership

In this commentary, we categorise peace and partnership as cross-cutting impacts as politicization [35], nationalism [41] and failure of global health governance [42] hampered pandemic containment and consequently impacted population, prosperity and planetary health. At the individual and household levels, COVID-19 has increased rates of suicide due to unemployment, homelessness, psychological consequences and domestic violence affecting children and women [23, 24]. The risks of violence, exploitation, and abuse might have increased due to economic uncertainty. COVID-19 responses can be abused by governments to undermine human rights such as using emergency laws to limit oppositions [43, 44].

Politicization of COVID-19 responses [45] and vaccine nationalism have been reported in various countries [46]. Vaccine nationalism means countries prioritize their domestic vaccine needs at the expense of others through advanced market commitment. On the one hand, it may accelerate efforts to develop such vaccines, but consequences outweigh this benefit; wealthy countries occupy the lion’s share of prospective doses for themselves and hamper global efforts for equitable vaccine distribution [47, 48]. Furthermore, India, the world’s largest producer of hydroxychloroquine, initially banned exporting hydroxychloroquine to protect domestic demand in the wake of the pandemic [49]. Vaccine nationalism is self-defeating. Though it benefits high income countries in the short term, they cannot contain local transmission due to arrival of international travellers from low vaccine coverage countries. Omicron variant identified in South Africa had spread worldwide rapidly [50]. This confirms the statement that “no country is safe, until all countries are safe” [51]*.* Only equitable vaccine distribution and high levels of coverage can limit the spread of new strains [52]. Donation of medical supplies and vaccines helps protect everyone and enhances international collaboration [46]. Rapid research and development of COVID-19 vaccines are the fruits of the global recognition of the need for international collaboration on vaccine development and roll-out, ensuring people in low- and middle- income countries (LMICs) have access to vaccines [53]. COVAX facility, a mechanism for vaccine internationalism which aim for equitable access of vaccines by all countries; however, faced significant challenges.

## Discussion

Analysis of literatures which guide the construction of Vensim® causal diagram resulted in five themes of impacts from pandemic. These are a) health of population, b) education of children, c) economic and employment, d) environment and e) food and nutrition. Based on 5Ps pillar, a)-c) focuses on People, d) focuses on Prosperity and e) focuses on Planet. This commentary applied a causal loop diagram to demonstrate the causes of COVID-19 and their amplified effects. The size of impacts depends on various determinants and the pre-existing vulnerability of populations of particular ethnicity. The UK report clearly demonstrated how pre-existing vulnerability resulted in larger adverse effects on the poor, minorities and other vulnerable populations [26].

This commentary demonstrated that the pandemic affects health, education, food and nutrition of the population as well as economic and environment. Clearly, people are the victims of the pandemic. Therefore, the discussion focuses on how to better manage pandemic containment and protect health of the population.

Universal health coverage (UHC) means ensuring equitable access to essential health services without the risk of financial catastrophes [54]. The current COVID-19 pandemic undoubtedly reaffirms the importance of UHC for effective response and intervention including testing, tracing, and treating everyone, particularly vulnerable people. Reports have shown negative association between UHC and service coverage index and COVID-19 cases and deaths (the higher the coverage index, the lower the cases and deaths from COVID-19) [55, 56]. Investing in UHC will support building back a better and fairer society during the recovery phase of this pandemic and prepare us for future health and environmental crises. The COVID-19 pandemic reinforces the importance of UHC and resilient health systems as crucial entry points in achieving the SDGs.

A robust and resilient health system is critical for effective responses to public health threats [57]. Fragile health systems cannot respond well to pandemics, which is detrimental to population health. Health systems resilience, robust public health function and the ability to mobilise surge capacity and adequate resources to support responses are essential for pandemic control and minimising morbidity and mortality. A study in 28 countries demonstrates four key attributes of effective responses: timely activation of comprehensive responses, adaptive health systems capacities, preservation of health systems functions and resources and reduction of vulnerability [58]. All governments need to strengthen preparedness and response capacity, especially through One Health approach. Surveillance of coronavirus in wildlife, especially bats [59], case-based surveillance systems, lab capacity to diagnosis novel viruses, and community-based contact tracing and quarantine are among key interventions. Governments also need to address the critical shortage of field epidemiologists and public health officers. Prevention and control of hospital infection will ensure patient safety and that healthcare facilities do not become sources of transmission.

Though the WHO six health systems building blocks, which include a) service delivery, b) health workforce, c) information, d) medical products, vaccines and technologies, e) financing, and f) leadership and governance, are essential, effective risk communication and community engagement and management of misinformation on social media are equally important in ensuring adherence to public health and social measures and vaccine acceptance [58]. Community engagement is considered core to all elements of health systems resilience [60]. Infodemic, the flood of information including false or misleading information, related to COVID-19 in digital and physical environments is a major threat. The spread of fake news and misinformation is fostered by the mistrust in governments, reduced acceptance of official information [61] and denial of vaccines. Trust is associated with implementation of government measures, public compliance, the effects of government action and low mortality rates [62].

Moreover, not only timely response to the pandemic, but also the policy trade-off between health, and the economy and governance contribute to COVID-19 cases and mortality in different countries [63, 64]. In many countries, continued economic activities were prioritized while postponing public health interventions [63]. Toggling between disease control and the economy may have costed more lives [65]. However, the governments have to balance between health of the people and economic recovery of the country based on size of epidemic [66]. Even though there is no doubt that travel restrictions and closure of businesses and factories are associated with economic downturn, quantifying the economic losses requires sophisticated methodologies [67]. The effective and timely responses to contain the pandemic require political leadership and evidence-based informed decisions [68]. In many countries, pandemic responses are complicated by politics, conflicts between federal and state governments, substandard central and local government relationship and multisectoral coordination, and sometimes poor governance.

## Conclusions

As demonstrated and reaffirmed by this commentary, “health is a precondition for and an outcome and indicator of all three dimensions of sustainable development” [69]. All 17 SDGs are interlinked; health systems, and health and wellbeing of the population are directly affected by the pandemic while impacts on the prosperity, education, planetary health, and food insecurity are indirect due to pandemic responses. COVID-19 has derailed progress towards the SDGs. We propose four-prong policy recommendations.

First, getting out of the acute phase of the pandemic requires high COVID-19 vaccine coverage, up to 70% in all countries to disrupt virus transmission, while maintaining high levels of face mask coverage and other personal protective behaviour. High-income countries should donate their vaccine surplus to low- and middle- income countries instead of providing a fourth dose when poorer countries have yet to administer first doses. It would be in the public health interest of every country, high income countries and LMIC.

Second, strides need to be made towards equitable recovery and mitigation of the impacts of the pandemic. Governments should provide universal social protection and income support to vulnerable populations such as the elderly, children, persons with disability, the homeless and migrants. In many countries, governments provide social transfers, such as social emergency funds in Portugal and Canada, cash payments to vulnerable households in Australia, and medical service accessibility in Korea [70]. Governments need to strengthen health systems resilience, increase preparedness capacity through One Health surveillance as coronavirus circulation in bats can be key sources of novel virus outbreaks. Continued commitment towards UHC in the light of fiscal constraints requires strong political leadership across different governments.

Third, misinformation and fake news weaken the public health response, undermine citizens’ adherence to public health measures and increase vaccine hesitancy. The government should manage the infodemic through working with communities, checking facts and introducing effective communication to combat the spread of misinformation [71]. Accountability and transparency around how government decisions are made in the pandemic response will regain trust and adherence of individuals towards control measures [72].

Finally, we suggest deep reflection by in-country stakeholders to draw lessons on pandemic control, which is determined by multiple factors, not only path dependence, but governance paradigms, political systems, cultural preferences, state capacity in mobilizing various socio-economic resources and politicized nature of risk management. In several countries, policy dilemma between health of population and economic impacts also influenced the pandemic outcomes [73] and excess mortality [74]. The pandemic significantly reshapes state-market-society relationship [63]. These reflections will guide how country strengthens health systems and community resilience to be ready for another major public health emergency.

## Data Availability

Not applicable for this study.

## Abbreviations

COVID-19: Coronavirus disease 2019
GDP: Gross domestic product
LDC: Least Developed Countries
LMICs: Low- and middle- income countries
LIC: Low- income countries
SDGs: Sustainable Development Goals
UHC: Universal health coverage
UN: United Nations
